# Contribution of Neuroinflammation to the Pathogenesis of Cancer Cachexia

**DOI:** 10.1155/2015/801685

**Published:** 2015-10-04

**Authors:** Alessio Molfino, Gianfranco Gioia, Filippo Rossi Fanelli, Alessandro Laviano

**Affiliations:** Department of Clinical Medicine, Sapienza University of Rome, Viale del Policlinico 155, 00161 Rome, Italy

## Abstract

Inflammation characterizes the course of acute and chronic diseases and is largely responsible for the metabolic and behavioral changes occurring during the clinical journey of patients. Robust data indicate that, during cancer, functional modifications within brain areas regulating energy homeostasis contribute to the onset of anorexia, reduced food intake, and increased catabolism of muscle mass and adipose tissue. In particular, functional changes are associated with increased hypothalamic concentration of proinflammatory cytokines, which suggests that neuroinflammation may represent the adaptive response of the brain to peripheral challenges, including tumor growth. Within this conceptual framework, the vagus nerve appears to be involved in conveying alert signals to the hypothalamus, whereas hypothalamic serotonin appears to contribute to triggering catabolic signals.

## 1. Introduction

Metabolic changes due to tumour growth profoundly impact nutritional status [1]. Anorexia and reduced food intake are frequently the presenting symptoms of several types of cancer [1, 2]. Although anorexia and reduced food intake largely contribute to weight loss of cancer patients, wasting cannot be accounted for by inadequate eating only. Indeed, cancer-induced derangement of protein, carbohydrate, and lipid metabolism magnifies the impact of anorexia on nutritional status and also reduces the efficacy of nutritional interventions [2].

Tumor-associated changes of energy and macronutrient metabolism, together with behavioral changes (i.e., anorexia and reduced food intake), negatively influence patients' quality of life and increase their morbidity and mortality [3]. Inflammation plays a major role in the pathogenesis of metabolic and behavioral abnormalities during disease. Consequently, inflammatory markers are frequently used as predictors not only of metabolic abnormalities but of clinical outcome as well. As an example, high circulating levels of C-reactive protein (CRP) are frequently observed in cancer patients with cachexia. Thus, CRP levels, in combination with reduced food intake and weight loss, could be used as a clinical marker of cancer cachexia. Moreover, CRP might be directly involved in cancer-related wasting since it has been shown to exacerbate tissue injury of ischemic necrosis in heart attack and stroke [4]. Therefore, a potential role for CRP in inflammatory conditions such as cancer could be speculated, in which increased CRP production leads to binding of CRP to exposed ligands in damaged cells, thereby increasing tissue injury [5]. Systemic inflammation is also correlated with increased proteasome-mediated proteolysis in skeletal muscle of cancer patients [6].

Cancer anorexia also appears to be significantly influenced by increased inflammatory status, as demonstrated by increased brain levels of proinflammatory cytokines such as interleukin-1 (IL-1) and tumor necrosis factor-*α* (TNF*α*) in experimental models of cancer anorexia [7–11]. In fact, blockade of circulating TNF or inhibition of intrahypothalamic interleukin-1 receptors enhances food intake in animal models of cancer anorexia [12, 13]. Proinflammatory cytokines alter brain neurochemistry by enhancing the release of neurotransmitters able to influence neuronal anorexigenic pathways such as serotonin [14]. Further supporting the role of increased inflammatory response in mediating the onset of anorexia, Jatoi et al. showed in a prospective, controlled, randomized trial that the percentage of cancer patients with appetite improvement was similar following eicosapentaenoic acid (EPA) supplementation or megestrol acetate intake, a potent appetite enhancer [15]. EPA is an omega-3 fatty acid whose biological effects include the modulation of inflammatory response. By competing with omega-6 fatty acids, EPA is degraded by cellular lipoxygenase and cyclooxygenase. However, the prostaglandins and leukotrienes deriving from the degradation of EPA exert less proinflammatory activities when compared to the prostaglandins and leukotrienes deriving from the degradation of omega-6 fatty acids. Therefore, reduced production of omega-6 fatty acid-derived mediators of inflammation through supplementation of pharmacological doses of omega-3 fatty acids is now considered to play a contributory role in reducing inflammation and promoting preservation of nutritional status in cancer patients [16].

## 2. Interaction between Neuroinflammation and Neurotransmission

**Figure 1:**
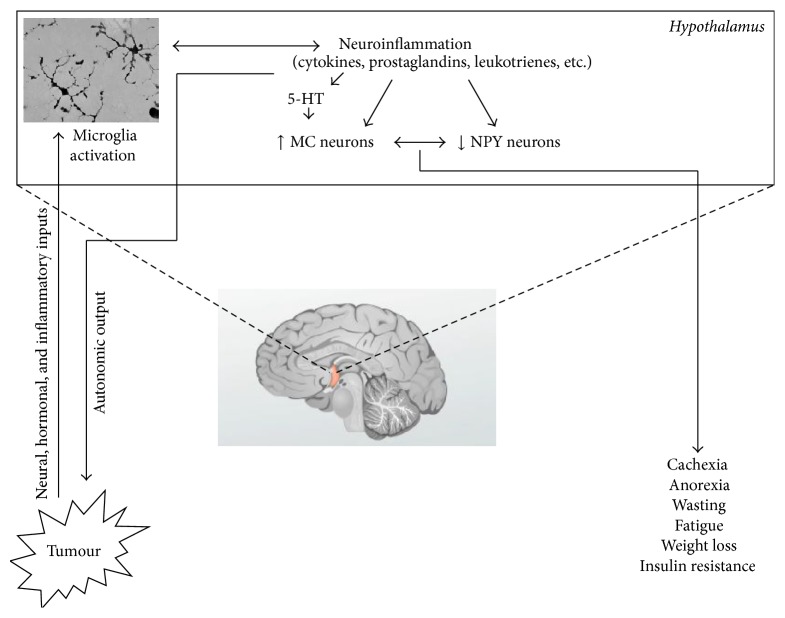
The growing tumor is sensed by the brain via neural, humoral, and inflammatory input. These signals activate the behavioural and metabolic response to stress by activating microglia cells, although it cannot be excluded that signals from peripheral tissues directly influence the activity of hypothalamic neurons, at least in the initial phase of the response to stress. Microglia activation triggers and perpetuates neuroinflammation, which is characterized by the release of inflammatory mediators within the hypothalamic areas. In the arcuate nucleus, inflammatory response hyperactivates catabolic neurons, that is, melanocortin (MC) neurons, which in turn contribute to the inhibition of prophagic neurons, that is, neuropeptide Y (NPY) neurons. Disruption of the physiological balance between the activity of MC and NPY neurons yields to the behavioural and metabolic consequences of cachexia. Experimental data also suggest that neuroinflammation may contribute to tumour growth and aggressiveness by modulating the peripheral immune response through autonomic output.

During cancer, the physiological functioning of the brain areas controlling energy homeostasis is disrupted. Consistent evidence indicates that increased hypothalamic expression and release of mediators of inflammation play a large role in this event (Figure 1). Proinflammatory cytokines such as IL-1 and TNF*α* have been recognized for many years as principal actors in the pathogenesis of anorexia and cachexia [17]. Hypothalamic IL-1 mRNA expression and IL-1 levels are significantly increased in the cerebrospinal fluid of anorexic tumor-bearing rats and inversely correlate with energy intake [7, 18]. The causative role of brain IL-1 in cancer anorexia and cachexia is supported by data showing that anorexia ameliorates after intrahypothalamic injection of the IL-1 receptor antagonist [19]. Intraperitoneal injection of recombinant human soluble TNF*α* receptor in experimental models improves anorexia thus confirming the role of TNF*α* in the negative modulation of appetite [12]. Finally, megestrol acetate, a potent orexigenic drug largely used in cancer patients, improves food intake by reducing the expression of IL-1 by mononuclear cells and by increasing hypothalamic concentrations of the prophagic mediator neuropeptide Y (NPY), which confirms the significant role of IL-1 in mediating cancer-associated anorexia in humans [20, 21].

Proinflammatory cytokines appear to exert their effects through their influence on the physiological hypothalamic pathway promoting catabolism, that is, the melanocortin system. Intracerebroventricular injection of IL-1 increases the frequency of signaling of melanocortin neurons in the arcuate nucleus of hypothalamus which express the type 1 IL-1 receptor. In addition, IL-1 stimulates the release of *α*-MSH [22]. Also, the classical neurotransmitter serotonin appears to be involved (Figure 1).

Serotonin contributes to energy balance by triggering satiety through its effects in the hypothalamus [23, 24]. Increased hypothalamic serotonin levels have been associated with the onset of cancer anorexia in experimental* in vivo *models and increased expression of serotonin receptors (5-HTRs). The link between serotonergic neurotransmission and disease-related anorexia is confirmed by the restoration of energy intake after tumor resection and normalization of hypothalamic serotonin concentrations and receptor expression [25, 26]. Intrahypothalamic injection of the serotonin antagonist mianserin ameliorates energy intake in experimental models of anorexia [13]. The synthesis of the hormone melatonin is determined by its precursor serotonin. Melatonin modulates the activity of the hypothalamic suprachiasmatic nucleus and alters biological rhythms. Disrupted melatonin synthesis and secretion in patients with cachexia and in wasted animals may contribute to serotonin accumulation in the hypothalamus [27, 28]. Serotonin plays a role in disease-associated anorexia, as confirmed by increased plasma and cerebrospinal fluid levels of the amino acid tryptophan, the precursor of serotonin, in anorexic and cachectic cancer patients [29]. Catabolic effects may be the consequence of the brain accumulation of tryptophan during the disease [30]. Brain tryptophan is also crucial in determining the release of kynurenine and its derivatives, molecules able to modulate immune functions [30]. Kynurenine represents the most important pathway, because tryptophan is mostly degraded via this pathway, producing 3-hydroxykynurenine and 3-hydroxyanthranilic acid, which represent acid free radical generators. The rate of tryptophan degradation through the kynurenine pathway is mediated directly by inflammation. In this light, the accumulation of brain tryptophan coupled with increased release of proinflammatory cytokines may maintain tryptophan metabolism toward increased free radicals production, determining enhanced oxidative stress. In experimental models of cancer-associated anorexia, increased concentrations of markers of oxidative stress have been measured in hypothalamic regions involved in the control of energy homeostasis [31].

As previously mentioned, melatonin biosynthetic pathway might be involved in the pathogenesis of anorexia. Melatonin exerts antioxidant function, and since the brain is largely composed of unsaturated fatty acids, preferential targets of reactive oxygen species, it could be speculated that melatonin supplementation may limit brain oxidation-induced inflammation and thus ameliorate anorexia and cachexia. However, Del Fabbro et al. have recently reported that oral melatonin 20 mg at night did not improve appetite, weight, or quality of life compared with placebo [32]. However, since the trial involved, among others, patients with gastrointestinal cancer, a role for the mechanical impact of tumor burden on the lack of clinical effects cannot be excluded.

## 3. The Melanocortin System and Its Role during Inflammation 

Melanocortin system mediates the anorectic effects of serotonin, as demonstrated by the activation of the central melanocortin pathway after the administration of fenfluramine, a serotonin reuptake inhibitor [33]. Studies have focused on 2 subtypes of serotonin receptors, the 5-HT2cR and the 5-HT1bR which are located within the arcuate nucleus of the hypothalamus. Anorexigenic neurons express 5-HT2cRs, whereas orexigenic NPY neurons express 5-HT1bRs. Agonists activate these receptors thus hyperpolarizing the NPY neurons while dramatically reducing the inhibitory postsynaptic potentials in melanocortin neurons [34]. An improvement in glucose tolerance and a decrease in plasma insulin levels were consequent to the administration of doses of 5-HT2cR agonists in experimental models of obesity via melanocortin-4 receptor signaling pathways [35]. Serotonin, IL-1, and TNF*α* are able to influence the activity of the central melanocortin system. In fact, peripheral infusion of IL-1 causes anorexia by increasing brain tryptophan levels and serotonin synthesis [36]. TNF*α* and IL-1 are able to regulate neuronal serotonin transporter [37]. Experimental data suggest that catabolic states are associated with increased hypothalamic expression of IL-1 together with enhanced release of serotonin. The function of the melanocortin system is conditioned by the interaction between serotonin and IL-1 within the arcuate nucleus. The consequences are the inhibition of NPY neuronal activity and the stopping of the inhibition of melanocortin neurons. These effects alter the melanocortin system by enhancing the release of *α*-MSH, an endogenous melanocortin receptor type 4 (MC4R) agonist, and suppressing the release of agouti-related peptide (AgRP), an endogenous MC4R antagonist. Interestingly, binding of *α*-MSH on MC1R reduces TNF*α* secretion by macrophages, therefore determining anti-inflammatory effects [38, 39].

The activation of the melanocortin system during peripheral acute stress is likely related to the direct sensing by hypothalamic cells of humoral or nervous triggers. However, during chronic stress, the role of neuroinflammation, and particularly of brain microglia, is key (Figure 1). The most important immune effector cells of the brain are microglia, the tissue macrophages of the brain, and they are involved in the onset, maintenance, relapse, and progression of brain inflammation. Under healthy conditions, microglia is characterized by a ramified morphology, which is used to continuously scan the environment. Upon any homeostatic disturbance, microglia rapidly change their phenotype and contribute to processes including inflammation, tissue remodeling, and neurogenesis. During activation, microglia releases neurotrophic factors, as well as neurotoxic factors and proinflammatory cytokines. Host defense is dependent on microglial activation, although detrimental effects have been also reported. However, robust and consistent evidence shows that microglia stimulates myelin repair, removal of toxic proteins, and prevention of neurodegeneration [40]. Recent data show that functional phenotypes of microglia differ according to the diverse brain regions and to the different types of stress (i.e., neuroinflammation, neurogenesis, brain tumour homeostasis, and aging) [41].

## 4. From Neuroinflammation to Systemic Inflammation 

Consistent evidence supports the concept that inflammation drives a multifactorial central and peripheral network of signaling pathways involved not only in the pathogenesis of cancer cachexia, but in tumor development and progression as well. In addition, inflammatory response is associated with increased circulating levels of specific cytokines, such as IL-1, IL-6, IFN*γ*, TNF*α* [42], and acute-phase proteins that lead to hypermetabolism and weight loss in patients with anorexia and cachexia [43]. Also, in advanced stages of cancer, IL-1*β* is strongly associated with loss of appetite, weight loss, sarcopenia, and general weakness [44]. Despite this robust evidence, it should be also acknowledged that Kayacan et al. did find increased concentrations of TNF*α* and IL-6 in patients with lung cancer, but they could not observe any significant difference between cachectic and noncachectic patients [45]. This highlights the importance of considering the circadian rhythm of cytokine production and release when measuring their circulating levels.

The mechanistic interaction between neuroinflammation, systemic inflammation, and tumor development has not yet been completely clarified. Evidences for a causal relationship between neuroinflammation and systemic inflammation and features of cachexia are increasing [46]. In models of anorexia and cachexia, administration of proinflammatory cytokines induced acute-phase protein response, anorexia, weight loss, protein and adipose tissue catabolism, and higher concentration of cortisol and glucagon, as well as decreased insulin resistance and a positive modulation of energy homeostasis [47]. In addition, high IL-6 levels correlated with cachexia phenotype, while treatment with monoclonal antibody to IL-6 reversed this picture [48]. When the specific role of neuroinflammation in the development and progression of cancer is considered (Figure 1), results obtained show that the sympathetic nervous system modulates the antitumor immune defense response. In fact, chemically sympathectomized tumor-bearing rats had significantly increased neutrophil-to-lymphocyte ratio, an indicator of disease progression, although no significant changes in tumor growth and survival were observed [49]. Also, Magnon et al. found that the formation of autonomic nerve fibers in the prostate gland regulates prostate cancer development and dissemination in mouse models. Moreover, a retrospective blinded analysis of prostate adenocarcinoma specimens from 43 patients revealed that the densities of sympathetic and parasympathetic nerve fibers in tumor and surrounding normal tissue, respectively, were associated with poor clinical outcomes [50]. Whether increased tumor innervation by autonomic nervous system could be regulated by increased brain inflammatory response remains to be ascertained. However, microglial activation has been demonstrated to contribute to the endocrine dysregulation and the elevated sympathetic nerve activity reported in streptozotocin-treated rats [51].

## 5. Brain and Muscle-Adipose Tissue Axis 

Robust data indicate that the control of energy intake and expenditure is largely mediated by the hypothalamus, and centrally produced proinflammatory cytokines participate in activating the molecular modifications inducing the development of cancer-associated anorexia and cachexia [46]. Moreover, experimental models of wasting showed that muscle catabolism during disease is activated by hypothalamic stimuli and cytokines may enhance the activity of the hypothalamic melanocortin system promoting muscle and adipose wasting [46].

The interaction between inflammatory mediators and the central nervous system may occur at the peripheral levels and may play a relevant role in triggering the host inflammatory response. This inflammatory response, when constantly present, may lead to the development of cachexia. At peripheral levels, tumour growth could be sensed by the vagus nerve, possibly by sensing the paracrine release of proinflammatory cytokines [52]. This information is conveyed to brainstem regions and finally to the hypothalamus, activating the melanocortin system through specific neural intermediates and receptors [53]. The melanocortin system, when activated, enhances the release of cytokines to reduce food intake and promote muscle catabolism. Consequently, inhibition of the brain inflammatory response that is induced by cytokines may result in better clinical outcome than systemic immune suppression. In this light, exploration of the possible pathogenic and clinical roles of fatty acid-derived modulators of inflammation may yield relevant results.

As previously mentioned, EPA supplementation contributes to anticachexia therapy by reducing inflammatory response. Docosahexaenoic acid (DHA) is the major brain omega-3 fatty acid and has been shown to be involved in the biosynthesis of potent anti-inflammatory and proresolving mediators by macrophages, maresins [54]. Although their biological function has been investigated in experimental models of acute inflammation, a possible role during clinical conditions characterized by mild to moderate, yet chronic, inflammatory response, including cancer, cannot be excluded. Greater relevance for the pathogenic link between neuroinflammation and cachexia appears to be exerted by neuroprotectins.

Similarly to maresins, DHA is the precursor of neuroprotectins as well [55]. Consistent evidence showed that neuroprotectins attenuate brain damage following ischemia and restore nerve integrity and function after experimental surgery. Also, neuroprotectin D1 has been shown to induce homeostatic regulation following proteotoxic stress induced by misfolding proteins [56]. Such type of stress appears more similar to that induced by a growing tumour and therefore suggests that neuroprotectins could be a relevant therapeutic target to specifically inhibit the brain contributory role to cachexia of cancer.

## 6. Conclusion 

During the last few years, our knowledge of the mechanisms regulating neural inflammation has been largely improved. However, the impact on clinical practice of these advancements in the pathophysiology of neuroinflammation and its link with systemic inflammation is still lacking. This may be determined by the heterogeneity of the symptoms characterizing anorexia and cachexia in human conditions. It is extremely likely that the different clinical conditions induced by inflammation are determined by the polymorphisms of different genetic profile [57], which in turn regulates the neurochemical/metabolic response to similar challenges. In this light, it appears mandatory to focus our research on the identification of polymorphisms of key genes, regulating the expression of inflammatory markers and possibly serotonin. This approach will allow the use of preventative or early anticatabolic therapies.
